# Increased risk of thrombosis in JAK2 V617F-positive patients with primary myelofibrosis and interaction of the mutation with the IPSS score

**DOI:** 10.1038/s41408-022-00743-0

**Published:** 2022-11-16

**Authors:** Tiziano Barbui, Arianna Ghirardi, Alessandra Carobbio, Arianna Masciulli, Greta Carioli, Alessandro Rambaldi, Maria Chiara Finazzi, Marta Bellini, Elisa Rumi, Daniele Vanni, Oscar Borsani, Francesco Passamonti, Barbara Mora, Marco Brociner, Paola Guglielmelli, Chiara Paoli, Alberto Alvarez-Larran, Ana Triguero, Marta Garrote, Helna Pettersson, Björn Andréasson, Giovanni Barosi, Alessandro Maria Vannucchi

**Affiliations:** 1 FROM Research Foundation, ASST Papa Giovanni XXIII, Bergamo, Italy; 2 ASST Papa Giovanni XXIII, Bergamo, Italy; 3 University of Milan, Milan, Italy; 4 Department of Molecular medicine, University of Pavia, Pavia, Italy; 5 Hematology, Fondazione IRCCS Policlinico San Matteo, Pavia, Italy; 6 University of Insubria, Varese, Italy; 7 ASST Sette Laghi, Varese, Italy; 8 CRIMM, Azienda Ospedaliera Universitaria Careggi, Florence, Italy; 9 University of Florence, Florence, Italy; 10 Hematology Department, Hospital Clínic, Barcelona, Spain; 11 Hematopathology Unit, Pathology Department, Hospital Clínic, Barcelona, Spain; 12 Division of Hematology, NU Hospital Group, Uddevalla, Sweden; 13 Center for the Study of Myelofibrosis, IRCCS Policlinico S. Matteo Foundation, Pavia, Italy

**Keywords:** Risk factors, Myeloproliferative disease

Dear Editor,

Thrombosis remains a major unmet need in polycythemia vera (PV), essential thrombocythemia (ET), and primary myelofibrosis (PMF), accounting for three to four-fold higher probability of risk compared to controls [1]. In PMF, this risk is poorly characterized given that, unlike PV and ET, death due to disease progression and/or evolution to leukemia represents a competing risk that significantly overwhelms that attributable to vascular complications [2–5].

We analyzed a large cohort of PMF patients enrolled in the European ERNEST registry with the aim to identify patients at higher risk in which anti-thrombotic prophylaxis could be suggested. Primary prophylaxis with aspirin is debated in PMF and, since thrombosis is not considered a major issue, some experts prescribe this agent only as a secondary prophylaxis after an arterial event [6].

The ERNEST project was launched in 2013 to prospectively enroll primary and post–polycythemia vera (post-PV) and post–essential thrombocythemia (post-ET) MF patients with the epidemiological goal of assuring reliability, representativeness and comparability of real-word data across international centers with expertise in the management of MF [7]. The project, promoted by the European *LeukemiaNet* (ELN), was coordinated by FROM - Research Foundation at Papa Giovanni XXIII Hospital in Bergamo (Italy) - and supported by Novartis through a research collaboration. From February 2013 to May 2014, the ERNEST registry enrolled 1292 MF-WHO diagnosed patients from 13 centers in 5 European countries. This manuscript reports analysis of a subset of 584 PMF patients for whom updated information until the end of 2020 were provided by 6 centers from Italy, Spain, Sweden. Prefibrotic myelofibrosis as well as post-ET/PV myelofibrosis were not included.

Descriptive statistics were used to summarize the characteristics of PMF patients at diagnosis. Each patient was classified according to baseline IPSS and followed from the date of PMF diagnosis until occurrence of thrombosis or censoring (last contact/study-end fixed at December, 31^th^ 2020), whichever came first. Lower and higher-risk IPSS categories were compared grouping together low and intermediate-1 risk categories and intermediate-2 and high-risk categories, respectively. Univariate and multivariable Fine and Gray’s competing risk models were fitted to estimate the association between characteristics of PMF patients and post-diagnosis thrombosis, considering death as a competing event, and estimated sub-distribution Hazard Ratios (sHRs) with the corresponding 95% CIs were reported. Cumulative incidence functions (CIF) of thrombosis were estimated using competing risk methods and compared over time using Gray’s test. The effect of cytoreductive drugs (i.e., hydroxyurea (HU) and ruxolitinib (Ruxo)) on thrombotic risk was investigated by adjusting for immortal time bias with the Mantel-Byar method [8]. Finally, the impact of post-diagnosis thrombosis on mortality was estimated by a multivariable Cox model including thrombosis as a time-dependent variable.

After a median follow up of 3.9 years (interquartile range: 1.8–8.2), a total of 61 out of 584 subjects (10.4%) experienced a post-diagnosis thrombosis, corresponding to 1.82% patients per year (pt/y). Of these, 30 (5.1%; rate 0.93% pt/y) were arterial and 31 (5.3%; rate 0.89% pt/y) venous events.

In univariate analysis (Supplementary Table 1S), factors that significantly distinguished patients with thrombosis were younger age, lower-risk IPSS and *JAK2* V617F mutation; in contrast, no difference in the frequency of *MPL* and *CALR* mutation or triple negative status, was recorded. Hemoglobin levels were higher in the group of patients with thrombosis while leukocyte and platelet counts were comparable. As might be expected, the proportion of patients treated with HU tended to be higher in cases who developed post-diagnosis thrombosis. Interestingly, pre-diagnosis arterial but not venous events were predictors of future events. In multivariable competing risk model, adjusted for previous history of thrombosis, hemoglobin levels and cytoreductive treatment, the category of lower-risk IPSS lost the statistical significance, even though a trend towards an increased risk of thrombosis was documented (sHR = 1.73, 95% CI 0.90–3.32); in contrast, *JAK2* mutation retained its prognostic value (sHR = 3.06, 95% CI 1.32–7.07) independently from the two IPSS groups (Fig. 1, panel A). By adjusting for immortal time bias, we failed to show an effect of HU (*n* = 272) and Ruxolitinib (*n* = 69) exposure on the risk of thrombosis (sHR = 1.14, 95% CI 0.65–1.99).

**Fig. 1:**
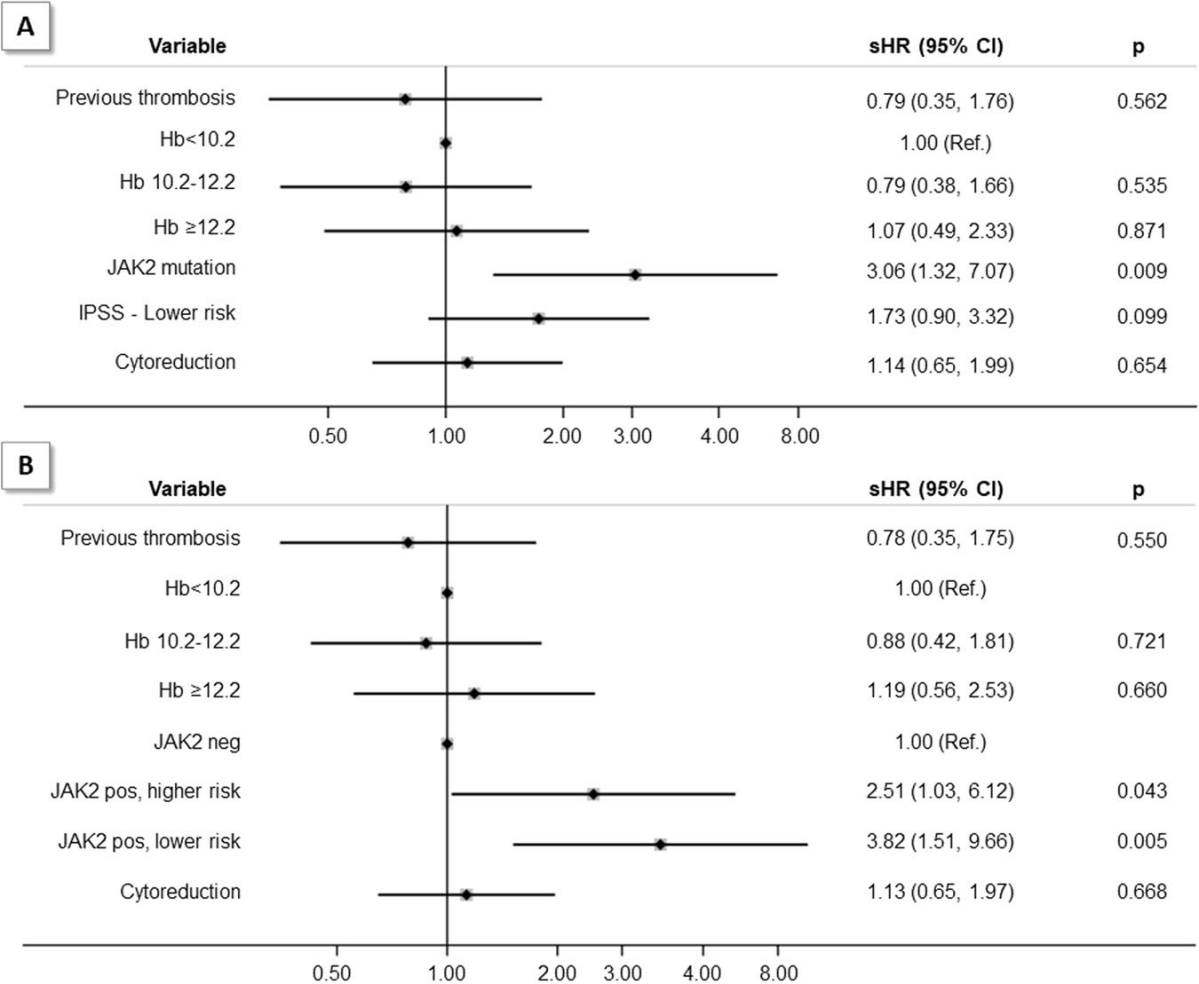
Predictors of post-diagnosis thrombosis estimated by a multivariable Fine and Gray’s competing risk model adjusted for potential confounders (death was considered as a competing event). sHR Sub-distribution Hazard Ratio, CI Confidence Interval. Hb Hemoglobin (g/dl). JAK2 pos, higher risk: patients with the combination JAK2 V617F mutated and intermediate-2/high IPSS. JAK2 pos, lower risk: patients with the combination JAK2 V617F mutated and low/intermediate -1 IPSS.

Few patients in this study received aspirin (*n* = 61) or oral anticoagulants (*n* = 39) as primary or secondary prophylaxis thus precluding efficacy analyzes; we report major hemorrhagic events after aspirin (*n* = 10) and oral anticoagulants (*n* = 4).

Figure 2 illustrates the cumulative incidence function (CIF) of thrombosis stratified by IPSS (panel A) and *JAK2* V617F mutation (panel B), considering death as a competing event. Cumulative incidence of thrombosis after 10 years for patients with lower-risk IPSS was similar to the group of *JAK2* V617F mutated patients (14% and 15%). However, in patients with lower vs. higher-risk IPSS, the cumulative incidence was substantially higher (*p* = 0.042) and the corresponding sHR was 1.73. The two groups did not differ for generic risk factors of thrombosis such as smoking (3% vs. 2%, *p* = ns), diabetes (20% vs. 18%, *p* = ns) and arterial hypertension (80% vs. 65%, *p* = ns).

**Fig. 2:**
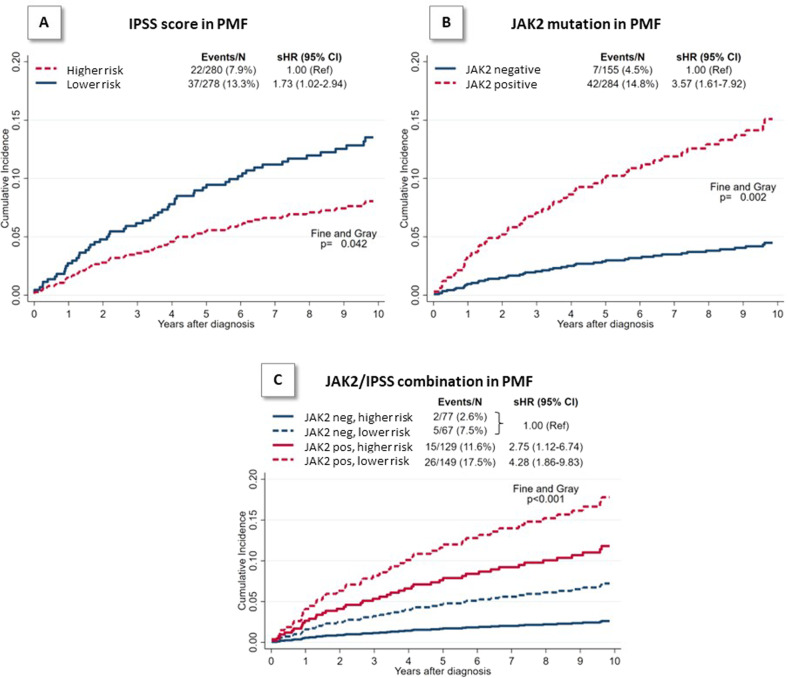
10-year Cumulative Incidence Function (CIF) of total thrombosis (death was considered as a competing event). Cumulative incidence of thrombosis was represented according to IPSS score (panel **A**), JAK2 mutation (panel **B**) and their combination (panel **C**) Lower risk = low and intermediate-1 IPSS categories. Higher risk = Intermediate-2 and high IPSS categories.

In patients with *JAK2 V617F* mutation the risk was over 3-fold higher than in un-mutated cases (sHR = 3.57, 95% CI 1.61–7.92) particularly in lower-risk IPSS patients (Fig. 2, panel C). In this latter subgroup, in comparison with *JAK2* unmutated patients, sHR was 4.28 (95% CI 1.86 –9.83) while for *JAK2* V617F mutated patients in higher-risk IPSS category was 2.75 (95% CI 1.12–6.74).

The combination of *JAK2 V617F* mutation and lower-risk IPSS was independently associated with thrombosis as shown in multivariable model by adjusting for prior thrombosis, hemoglobin levels and cytoreductive treatment (Fig. 1, panel B). We speculate that the reason for this finding may be related to most proliferative condition in *JAK2* mutated lower-risk IPSS patients as shown by the normal hemoglobin levels and significantly higher platelet counts (*p* = 0.001) with respect to *JAK2* mutated patients with higher-risk IPSS (Supplementary Table 2S). No difference for other indicators of myeloproliferation, such as splenomegaly or *JAK2* V617F allele burden, was found.

These findings are confirmed in *JAK2* unmutated patients in which the likelihood of thrombosis after 10 years tended to be higher among patients included in the lower (7%) than higher-risk IPSS category (3%) (*p* = 0.203).

The effect of the JAK2/IPSS combination on the risk of thrombosis was confirmed in an external cohort of patients with PMF (*N* = 380) that included 115 patients with *JAK2* V617F and lower-risk IPSS (30%), in which the cumulative incidence of thrombosis was 15% after 10 years. Conversely, in *JAK2* mutated and higher-risk IPSS the CIF was 9% (Dr. Barosi, Pavia, Italy).

By adjusting for age, gender, IPSS and cytoreduction, thrombosis had an independent effect on the risk of death (HR = 1.78, 95% CI 1.23–2.56). The 40 fatal thrombotic events occurred simultaneously with other complications leading to death (i.e., MF progression (9, 22.5%), infections (3, 7.5%), bone marrow transplant toxicities (1, 2.5%), unknown causes (60%)); in 3 patients only (7.5%) thrombosis was assigned to cause death.

The strengths of this study are the large cohort of PMF patients followed for a median time of 3.9 years, the number of thrombotic events and the external validation of the main results. The weaknesses are linked to its retrospective design with possibility of bias regarding patient selection and missing data, particularly on aspirin primary prophylaxis.

In summary, we provided evidence that PMF patients have a relevant and life-threatening risk of thrombosis and that the lower-risk IPSS categories (low/intermediate-1) harboring the *JAK2 V617F* mutation are at the highest risk. In clinical practice, this risk could justify studies testing primary prophylaxis with low-dose aspirin in all positive *JAK2 V617F* patients, particularly in those with lower-risk IPSS scores, if not otherwise contraindicated.

## Supplementary information

Table 1S

Table 2S

## Data Availability

Aggregated data available by request. Patient-level data will not be shared.
