# Supplementary material for: Increased risk of thrombosis in JAK2 V617F-positive patients with primary myelofibrosis and interaction of the mutation with the IPSS score
Source: Blood Cancer J. 2022 Nov 16;12(11):156. doi: 10.1038/s41408-022-00743-0 (PMC9668822; doi:10.1038/s41408-022-00743-0)
Supplement: Supplementary file 1 — Table 1S [file 41408_2022_743_MOESM1_ESM.docx]

**SUPPLEMENTAL MATERIAL**

**Table 1S. Patients’ demographics and clinical characteristics at diagnosis for patients with/without thrombotic events after MF diagnosis**

|  | **Total** | **THROMBOSIS** | | **p*** | **sHR (95% CI)** | **p**** |
| --- | --- | --- | --- | --- | --- | --- |
|  | **N=584** | **NO (N=523)** | **YES (N=61)** |  |  |  |
| **Age**, mean (SD) | 63.7 (13.1) | 64.1 (13.1) | 60.2 (12.4) | 0.028 | 0.98 (0.97-1.00) | 0.016 |
| **Male,** *n (%)* | 379/584 (64.9) | 337/523 (64.4) | 42/61 (68.9) | 0.49 | 1.22 (0.71-2.09) | 0.47 |
| **IPSS score,** *n (%)* |  |  |  |  |  |  |
| Lower risk | 278/558 (49.8) | 241/499 (48.3) | 37/59 (62.7) | 0.036 | 1.73 (1.02-2.94) | 0.042 |
| Higher risk | 280/558 (50.2) | 258/499 (51.7) | 22/59 (37.3) |  | 1.00 (Ref). | - |
| **Palpable spleen,** *n (%)* |  |  |  |  |  |  |
| No | 93/465 (20.0) | 82/416 (19.7) | 11/49 (22.4) | 0.65 | 1.00 (Ref). | - |
| Yes | 372/465 (80.0) | 334/416 (80.3) | 38/49 (77.6) |  | 0.87 (0.44-1.72) | 0.69 |
| **Spleen length (cm below costal margin),** *n (%)* |  |  |  |  |  |  |
| < 5 cm | 177/349 (50.7) | 159/312 (51.0) | 18/37 (48.6) | 0.013 | 1.00 (Ref). | - |
| 5-10 cm | 103/349 (29.5) | 86/312 (27.6) | 17/37 (45.9) |  | 1.74 (0.90-3.36) | 0.10 |
| ≥ 10 cm | 69/349 (19.8) | 67/312 (21.5) | 2/37 (5.4) |  | 0.28 (0.07-1.20) | 0.087 |
| **Symptomatic disease at diagnosis,** *n (%)* |  |  |  |  |  |  |
| No | 289/548 (52.7) | 257/491 (52.3) | 32/57 (56.1) | 0.67 | 1.00 (Ref). | - |
| Yes | 259/548 (47.3) | 234/491 (47.7) | 25/57 (43.9) |  | 0.88 (0.52-1.49) | 0.63 |
| ***Driver mutations, n (%)*** |  |  |  |  |  |  |
| *JAK2* V617F | 284/439 (64.7) | 242/390 (62.1) | 42/49 (85.7) | 0.001 | 3.57 (1.61-7.94) | 0.002 |
| *Homozygote* | 46/154 (29.9) | 42/132 (31.8) | 4/22 (18.2) | 0.31 | 0.51 (0.17-1.52) | 0.22 |
| *Heterozygote* | 108/154 (70.1) | 90/132 (68.2) | 18/22 (81.8) |  | 1.98 (0.66-5.95) | 0.22 |
| *MPL* W515 | 22/322 (6.8) | 19/287 (6.6) | 3/35 (8.6) | 0.72 | 1.22 (0.40-3.75) | 0.73 |
| *CALR* | 44/353 (12.5) | 42/307 (13.7) | 2/46 (4.3) | 0.093 | 0.29 (0.07-1.19) | 0.086 |
| *Type 1* | 33/353 (9.3) | 32/307 (10.4) | 1/46 (2.2) | 0.100 | 0.20 (0.03-1.45) | 0.11 |
| *Type 2* | 11/352 (3.1) | 10/306 (3.3) | 1/46 (2.2) | 1.00 | 0.62 (0.09-4.28) | 0.62 |
| **Triple-negative** | 22/164 (13.4) | 21/143 (14.7) | 1/21 (4.8) | 0.31 | 0.30 (0.04-2.36) | 0.25 |
| **Lab values** |  |  |  |  |  |  |
| **Hemoglobin (g/dL),** *median (IQR)* | 11.0 (9.4-12.7) | 11.0 (9.4-12.6) | 12.0 (10.1-13.7) | 0.031 | 1.10 (0.99-1.22) | 0.063 |
| < 10.2 | 209/559 (37.4) | 194/500 (38.8) | 15/59 (25.4) | 0.045 | 1.00 (Ref.) | - |
| 10.2-12.2 | 177/559 (31.7) | 159/500 (31.8) | 18/59 (30.5) |  | 1.39 (0.70-2.74) | 0.35 |
| ≥ 12.2 | 173/559 (30.9) | 147/500 (29.4) | 26/59 (44.1) |  | 2.13 (1.13-4.03) | 0.02 |
| **WBC count (x 10^9^/L),** *median (IQR)* | 8.9 (5.8-14.2) | 8.9 (5.7-14.3) | 9.0 (6.2-13.8) | 0.87 | 1.00 (0.98-1.02) | 0.89 |
| < 7.21 | 200/557 (35.9) | 180/498 (36.1) | 20/59 (33.9) | 0.83 | 1.00 (Ref.) | - |
| 7.21-12.75 | 197/557 (35.4) | 174/498 (34.9) | 23/59 (39.0) |  | 1.18 (0.65-2.13) | 0.59 |
| ≥ 12.75 | 160/557 (28.7) | 144/498 (28.9) | 16/59 (27.1) |  | 0.99 (0.51-1.91) | 0.97 |
| **Platelets (x 10^9^/L),** *median (IQR)* | 273.0 (148.5-530.0) | 271.0 (148.0-528.0) | 306.0 (167.0-620.0) | 0.39 | 1.00 (1.00-1.00) | 0.72 |
| < 206.5 | 206/556 (37.1) | 187/497 (37.6) | 19/59 (32.2) | 0.64 | 1.00 (Ref.) | - |
| 206.5-438.5 | 172/556 (30.9) | 151/497 (30.4) | 21/59 (35.6) |  | 1.31 (0.71-2.45) | 0.39 |
| ≥ 438.5 | 178/556 (32.0) | 159/497 (32.0) | 19/59 (32.2) |  | 1.12 (0.60-2.12) | 0.72 |
| **History of thrombosis before MF diagnosis** |  |  |  |  |  |  |
| **Previous thrombosis** | 88/584 (15.1) | 78/523 (14.9) | 10/61 (16.4) | 0.76 | 1.11 (0.56-2.20) | 0.76 |
| Arterial | 59/88 (67.0) | 51/78 (65.4) | 8/10 (80.0) | 0.039 | - | - |
| Venous/Splanchic | 28/88 (31.8) | 27/78 (34.6) | 1/10 (10.0) |  | - | - |
| Arterial & Venous | 1/88 (1.1) | 0/78 (0.0) | 1/10 (10.0) |  | - | - |
| **Treatments during follow-up** |  |  |  |  |  |  |
| **Cytoreduction** |  |  |  |  |  |  |
| No | 243/584 (41.6) | 224/523 (42.8) | 19/61 (31.1) | 0.080 | 1.00 (Ref.) | - |
| Yes | 341/584 (58.4) | 299/523 (57.2) | 42/61 (68.9) |  | 1.53 (0.89-2.64) | 0.12 |
| ***Type:*** |  |  |  |  |  |  |
| *Only HU* | 272/584 (46.6) | 236/523 (45.1) | 36/61 (59.0) | 0.24 | 1.67 (0.96-2.92) | 0.071 |
| *Ruxo after HU* | 39/584 (6.7) | 36/523 (6.9) | 3/61 (4.9) |  | 0.90 (0.27-2.98) | 0.86 |
| *Ruxo only* | 30/584 (5.1) | 27/523 (5.2) | 3/61 (4.9) |  | 1.21 (0.37-3.95) | 0.76 |
| ***Aspirin†*** | 66/82 (80.5) | 55/69 (79.7) | 11/13 (84.6) | 0.68 | 1.37 (0.29-6.44) | 0.69 |

*sHR: Sub-distribution Hazard Ratio; CI: Confidence Interval*

**According to the t-test or the Mann–Whitney U test for continuous variables and to the chi-square test (or Fisher’s exact test when appropriated) for categorical variables*

***According to the Fine and Gray competing risk model (death as competing event)*

*†Information on aspirin is only available for 82 patients over the total of 584 PMF patients*
