# Supplementary material for: Increased risk of thrombosis in JAK2 V617F-positive patients with primary myelofibrosis and interaction of the mutation with the IPSS score
Source: Blood Cancer J. 2022 Nov 16;12(11):156. doi: 10.1038/s41408-022-00743-0 (PMC9668822; doi:10.1038/s41408-022-00743-0)
Supplement: Supplementary file 2 — Table 2S [file 41408_2022_743_MOESM2_ESM.docx]

**SUPPLEMENTAL MATERIAL**

**Table 2S. Patients’ demographics and clinical characteristics at diagnosis for *JAK2* V617F mutated patients with PMF diagnosis according to IPSS categories**

|  | ***JAK2* V617F mutated patients with PMF** | | |
| --- | --- | --- | --- |
|  | **Lower risk** | **Higher risk** | **p*** |
|  | **N=149** | **N=129** |  |
| **Age**, mean (SD) | 60.1 (13.2) | 70.0 (9.2) | <0.001 |
| **Male,** *n (%)* | 100/149 (67.1) | 82/129 (63.6) | 0.53 |
| **Previous thrombosis***, n (%)* | 26/149 (17.5) | 22/129 (17.1) | 0.93 |
| **Hemoglobin (g/dL),** *median (IQR)* | 12.3 (11.2-14.1) | 9.9 (8.6-12.1) | <0.001 |
| < 10.2 | 20/148 (13.5) | 72/129 (55.8) |  |
| 10.2-12.2 | 50/148 (33.8) | 26/129 (20.2) | <0.001 |
| ≥ 12.2 | 78/148 (52.7) | 31/129 (24.0) |  |
| **WBC count (x 10^9^/L),** *median (IQR)* | 9.6 (7.0-13.9) | 9.0 (5.7-19.8) | 0.81 |
| **Platelets (x 10^9^/L),** *median (IQR)* | 345.0 (188.5-627.0) | 252.5 (148.5-420.0) | 0.001 |
| < 206.5 | 41/148 (27.7) | 50/128 (39.1) | 0.011 |
| 206.5-438.5 | 48/148 (32.4) | 48/128 (37.5) |  |
| ≥ 438.5 | 59/148 (39.9) | 30/128 (23.4) |  |
| **Cytoreduction during follow-up** | 105/149 (70.5) | 74/129 (57.4) | 0.023 |
| ***Type:*** |  |  |  |
| *Only HU* | 75/149 (50.3) | 57/129 (44.2) | 0.12 |
| *Ruxo after HU* | 15/149 (10.1) | 9/129 (7.0) |  |
| *Ruxo only* | 15/149 (10.1) | 8/129 (6.2) |  |
| ***Antiplatelets* during follow-up*** | 17/19 (89.5) | 20/23 (87.0) | 0.80 |

**According to t-test for continuous variables and the chi-square test (or Fisher’s exact test when appropriated) for categorical variables*

*†Information on antiplatelets was missing for 502 out of 584 PMF patients*
